# Extended surgical resection for nonfunctioning duodenal neuroendocrine tumor

**DOI:** 10.1093/jscr/rjac391

**Published:** 2022-09-06

**Authors:** Giorgio Lucandri, Giulia Fiori, Sara Lucchese, Vito Pende, Massimo Farina, Marco Giordano, Emanuele Santoro

**Affiliations:** 1st Department of Surgery, San Giovanni-Addolorata Hospital, Rome, Italy; 1st Department of Surgery, San Giovanni-Addolorata Hospital, Rome, Italy; 1st Department of Surgery, San Giovanni-Addolorata Hospital, Rome, Italy; 1st Department of Surgery, San Giovanni-Addolorata Hospital, Rome, Italy; 1st Department of Surgery, San Giovanni-Addolorata Hospital, Rome, Italy; Department of Pathology, San Giovanni-Addolorata Hospital, Rome, Italy; 1st Department of Surgery, San Giovanni-Addolorata Hospital, Rome, Italy

**Keywords:** neuroendocrine tumors, duodenum, pancreaticoduodenectomy, gastroenteropancreatic neuroendocrine tumor

## Abstract

Duodenal neuroendocrine tumors (NETs) account for <3% of all gastrointestinal NET. Most lesions are small-sized and are located in the first or second duodenal part. Tumoral grading, evaluated by Ki67 index, strongly influences patient’s outcome. Endoscopic resection is recommended for lesions measuring <2 cm, while pancreaticoduodenectomy should be the treatment of choice for large duodenal NET; Whipple procedure should be preferred in case of duodenal origin and contiguity with gastric antrum. Involvement of surrounding structures, as well as the presence of resectable liver metastases, does not contraindicate surgical resection. Herein we report a case of a 68-year-old male, presenting with an extensive mass of the descending pre-ampullary duodenal part, with involvement of the right colon and the presence of a pericholecystic single liver metastasis. In spite of such advanced disease, surgery on the patient was successful, with an uneventful postoperative outcome.

## INTRODUCTION

Duodenal neuroendocrine tumors (NETs) are relatively rare (accounting for 3% of all duodenal malignancies); they are classified according to different hormonal content, with about 28% of all NETs being nonfunctional. More than 90% of all duodenal NETs arise in the first and second parts of the duodenum [1,2]. Nodal metastases are present in 40–60% of cases, while synchronous liver metastases occur in 10% of all patients. They are present mainly in the sixth decade, with a slight male predominance. Usually, these tumors are discovered incidentally or present with nonspecific abdominal symptoms [1,3].

World Health Organization (WHO) 2019 NET grading classification is closely related to tumoral behavior and strongly influences patient’s prognosis [4]: as Ki67 Index and mitotic count increase, prognosis becomes worse (G3), even in the absence of nodal or liver metastatic disease [5].

**Figure 1:**
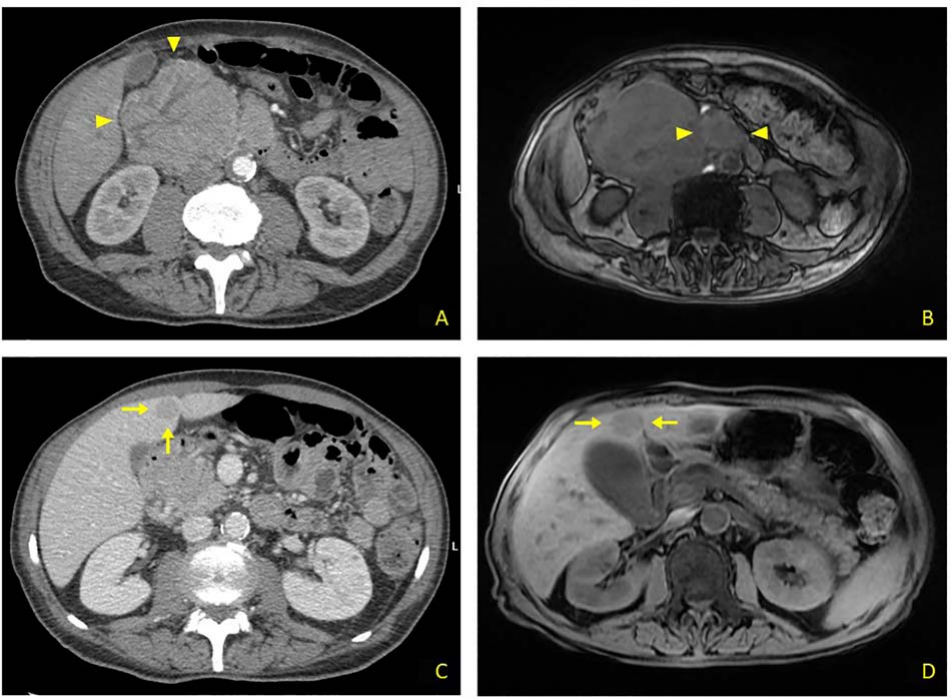
(**A**) Contrast CT scan: bulky formation with inhomogeneous uptake, arising from upper duodenal-pancreatic angle (arrowheads). (**B**) MRI: main lesion appears hypointense on fat-suppressed T1-weighted sequences. Evidence for further retromesenteric contrast enhanced tissue (arrowheads). (**C**) Contrast CT scan: hypodense rounded liver metastasis arising from segment V, measuring 2.5 cm in diameter (arrows). (**D**) MRI: T1-appearance of subglissonian pericholecystic liver metastasis (arrows).

Alteration in biomarker levels, such as serum chromogranin A and neuron-specific enolase, provides information on diagnosis and prognosis; upper endoscopy with biopsies and endoscopic ultrasound confirm diagnosis and degree of local spreading [6]. Current imaging modalities include both computed tomography (CT) and magnetic resonance imaging (MRI) [7]. Somatostatin receptor analogue scintigraphy in combination with CT (positron emission tomography [PET]-CT) is mandatory to detect small lymph nodes or liver/bone/extra-abdominal metastases [8]. Once the diagnosis of duodenal NET is confirmed, the most appropriate treatment depends on the patient’s Karnofsky Performance Status (KPS), tumor staging and grading.

**Figure 2:**
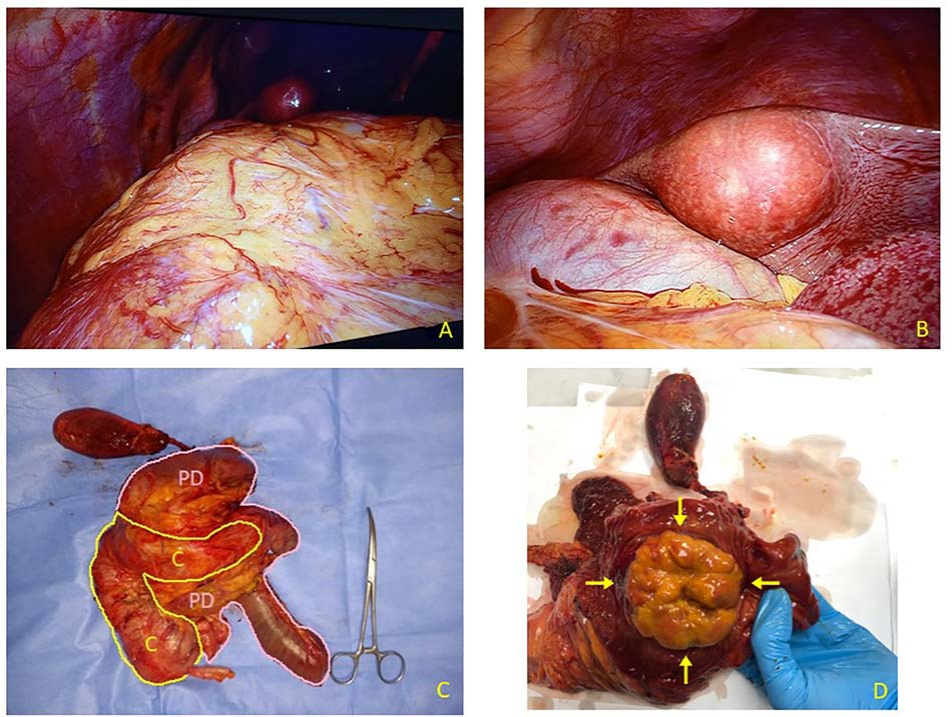
(**A**) Laparoscopic appearance of voluminous mass occupying right flank and hypochondrium. (**B**) Laparoscopic view of round-shaped pericholecystic liver metastasis. (**C**) Resected specimen includes right colon (C) and Whipple pancreatoduodenectomy (PD). Liver specimen has been examined separately. (**D**) After duodenal section, evidence for large rounded mass arising from descending pre-ampullary duodenal part (arrows).

**Figure 3:**
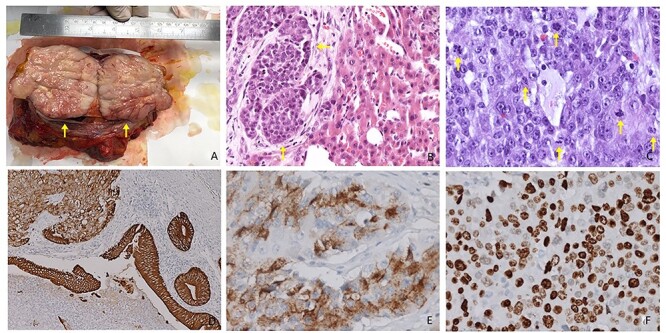
(**A**) Resected specimen: cut surface appears as firm, fleshy and pink to grey in color (arrows). (**B**) Hematoxylin and eosin staining. Morphological aspect of polygonal cells with oval nuclei, rounded nucleoli and abundant pale cytoplasm. Evidence for alveolar growth pattern (arrows, 20× magnification). (**C**) High-degree NET (G3) evidence for several mitotic figures (arrows) Mitotic Index: >20 × 2 mm^2^ (40× magnification). (**D**) IHC duodenal slide showing diffuse positivity for CK7 (10× magnification). (**E**) IHC slide showing diffuse positivity for chromogranin A (40× magnification). (**F**) IHC slide demonstrating a strong staining for Synaptophysin (40× magnification).

## CASE REPORT

A 68-year-old male was admitted to our Ward Unit reporting dyspepsia, weakness with weight loss and abdominal pain. Neither previous abdominal surgery nor a family history of cancer was referred. Patient complained of pain on the right flank (4.5 points in the Pain Scale Chart); canalization and food intake were described as regular. A large mass occupying the right abdomen was easily detected. Basic laboratory panel revealed anemia (Hb 9.8 g/dl) without leukocytosis or jaundice. Body mass index was 19.5 and KPS 70%. AngioCT revealed a neoplasm measuring 11 × 8.5 × 8 cm arising from the upper duodenal-pancreatic angle (Fig. 1A). Endoscopy confirmed its duodenal origin, but biopsies were not diagnostic. Prohance® MRI showed possible involvement of right colon and root of transverse mesocolon, with evidence of retromesenteric contrast enhanced tissue (Fig. 1B). Single liver metastasis, 2.5 cm in diameter, arising from segment V, was found (Fig 1C and D). Panel of tumoral markers showed increased values of chromogranin A and neuron-specific enolase, suggesting a diagnosis of nonfunctioning duodenal NET. Procedure-related morbidity contraindicated a percutaneous biopsy, hence diagnostic laparoscopy was performed: it confirmed the absence of peritoneal seeding and allowed a percutaneous 16G Tru-cut biopsy both for tumoral bulk and liver metastasis (Fig. 2A and B). Both samples showed a high-degree NET (G3). A 68Ga-DOTATOC PET/CT excluded any extra-abdominal disease, so the multidisciplinary board authorized a direct surgical approach.

After bilateral subcostal incision, both ascending colon and root of transverse mesocolon appeared infiltrated, while Kocher’s maneuver and dissection of hepatic pedicle confirmed no involvement of main vessels. Given the duodenal origin of the NET and contiguity with gastric antrum, a typical Whipple procedure was preferred; both right-sided hemicolectomy and atypical segment V liver resection were associated to main procedure (Fig. 2C). Soft pancreatic tissue and a normal-sized pancreatic duct (<2 mm) led us to consider pancreaticogastrostomy, so the pancreatic stump was inserted into posterior gastric wall. Both pancreaticogastrostomy and end-to-side hepaticojejunostomy were protected using a 15 cm Ch8 Bracci Wirutan® stent tube; end-to-side gastrojejunostomy and side-to-side ileotransversostomy were performed. Two left peripancreatic and one right subhepatic 4 × 10 mm Jackson-Pratt® drainages were positioned. The surgical operation lasted 345 min and estimated blood loss was 335 ml. The resected specimen weighed 1125 g, and after a section of the duodenal wall, a largely exophytic, rounded, pale-colored mass, measuring 12 × 10 cm, was detected (Fig. 2D).

The postoperative course was uneventful (Clavien-Dindo grade 1); during ICU stay, the patient received two blood transfusions and started enteral feeding. He was transferred to our ward on postoperative day (POD) 5; maximum amylase level in the drainage was 725 IU/l on POD 3, without any signs of clinical activity (Grade-A Pancreatic Fistula). Bowel movement started on POD 4 and oral intake on POD 7; drains were removed between POD 8 and POD 10 and patient was discharged on POD 13. At pathological examination, the lesion appeared rounded and multinodular in shape, without cleavage between pancreatic head and ascending colon; cut surface was firm, fleshy and pink to grey in color (Fig 3A). Histologic hematoxylin and eosin stain confirmed the presence of duodenal G3 NET (WHO 2019), with several mitotic figures and images of necrosis (Fig. 3B); Ki67 Labeling Index was >90%, with more than 20 mitoses per 10 high-power field (Fig. 3C). Liver metastasis shared similar histological features. Immunohistochemical panel confirmed positivity for CK7, CgA and Synaptophysin (Fig. 3D–F), while no metastases were found on 34 resected nodes. The tumor was staged as pT4 pN0 pM1a R0. Given this advanced staging with a high degree of malignancy and tumor’s nonfunctioning behavior, we recommended a protocol of early treatment with first-line systemic chemotherapy (Carboplatin plus Etoposide). The patient did not develop significant side effects and does not exhibit any clinical or radiological signs of recurrence or metastatic disease at 10 months since surgical treatment.

## DISCUSSION

Pancreaticoduodenectomy should represent the treatment of choice for large duodenal NET, with Whipple’s procedure preferred to pylorus preserving techniques given duodenal origin [9]. Involvement of surrounding organs, as well as the presence of resectable liver metastases, does not contraindicate surgical resection [10]. Less invasive surgical options should be reserved for selected cases and may have lesser long-term sequelae [11]. Prognostic value of nodal involvement still remains uncertain and the absence of definitive data led us to perform an extended nodal dissection (pN0_0/34_).

Endoscopic resection is recommended for lesions measuring <2 cm [12]. Tissue of origin does not seem to influence patient’s prognosis, which largely depends on tumor staging and grading [13–15]. In the patient we treated, the presence of nonfunctional NET with liver metastatic disease and a Ki67 Index >90% led us to suggest early adjuvant treatment with systemic chemotherapy and a close follow-up regimen.
